# The Relevance of Genomic Epidemiology for Control of Tuberculosis in West Africa

**DOI:** 10.3389/fpubh.2021.706651

**Published:** 2021-07-23

**Authors:** Prince Asare, Adwoa Asante-Poku, Stephen Osei-Wusu, Isaac Darko Otchere, Dorothy Yeboah-Manu

**Affiliations:** College of Health Sciences, Noguchi Memorial Institute for Medical Research, University of Ghana, Accra, Ghana

**Keywords:** genomic epidemiology, tuberculosis, West Africa, *Mycobacterium tuberculosis* complex, *Mycobacterium africanum*, tuberculosis control

## Abstract

Tuberculosis (TB), an airborne infectious disease caused by *Mycobacterium tuberculosis* complex (MTBC), remains a global health problem. West Africa has a unique epidemiology of TB that is characterized by medium- to high-prevalence. Moreover, the geographical restriction of *M. africanum* to the sub-region makes West Africa have an extra burden to deal with a two-in-one pathogen. The region is also burdened with low case detection, late reporting, poor treatment adherence leading to development of drug resistance and relapse. Sporadic studies conducted within the subregion report higher burden of drug resistant TB (DRTB) than previously thought. The need for more sensitive and robust tools for routine surveillance as well as to understand the mechanisms of DRTB and transmission dynamics for the design of effective control tools, cannot be overemphasized. The advancement in molecular biology tools including traditional fingerprinting and next generation sequencing (NGS) technologies offer reliable tools for genomic epidemiology. Genomic epidemiology provides in-depth insight of the nature of pathogens, circulating strains and their spread as well as prompt detection of the emergence of new strains. It also offers the opportunity to monitor treatment and evaluate interventions. Furthermore, genomic epidemiology can be used to understand potential emergence and spread of drug resistant strains and resistance mechanisms allowing the design of simple but rapid tools. In this review, we will describe the local epidemiology of MTBC, highlight past and current investigations toward understanding their biology and spread as well as discuss the relevance of genomic epidemiology studies to TB control in West Africa.

## Introduction and Background

### Tuberculosis Historical Trends and Current Burden

Tuberculosis (TB) is a disease of antiquity and eradication of it has been man's dream throughout history. Before the 19th century, very little was known about the causative pathogen and disease mechanisms. During the 17th to 19th centuries, reports indicated that 1 in every 5 adults had TB and mortality was 900 deaths per 100,000 population in the western world. TB accounted for 20% of all human deaths at the time (1). The history and perspective of TB was changed dramatically on March 24, 1882, with presentation by Robert Koch titled, *Die Aetiologie der Tuberkulose*, to the Berlin Physiological Society where Dr. Koch demonstrated the etiology of the disease and presented *Mycobacterium tuberculosis* as the causative agent (2).

The identification of the causative pathogen paved the way for several studies aimed at understanding the biology and the development of control tools including therapy. Antimycobacterial treatment began with the isolation of streptomycin (first isolated from *Streptomyces griseus* in 1944 by Albert Schatz, Elizabeth Bugie and Selman Waksman) followed in the 1950s and 60s by isoniazid and rifampicin (1). Nevertheless, TB persisted and does remain one of the leading causes of death among adults by a single infectious disease. TB affects millions of people annually so much so that in 1993 it became the first infectious disease to be declared a global health emergency by the World Health Organization (WHO). According to WHO estimates, about a quarter of the world's population are latently infected with the causative microorganism (3), thus, creating a pool of future active cases. Globally, in 2019 alone, an estimated 10 million new TB cases occurred, out of which 1.4 million died of TB making TB still the number one infectious disease killer by a single agent (4). Although the WHO African region is home to only 14% of the world's population, in 2019 it reported a quarter (25%, 2,460,000) of the global TB incidence, and currently has the highest HIV-associated TB cases and case fatality rates (4). This makes sub-Saharan Africa the most burdened region based on case to population ratio. Three of the 17 West African countries (Nigeria, Liberia, and Sierra Leone) are among WHO's list of 30 high TB burden countries globally. In addition, Nigeria, Liberia, Ghana, and Guinea Bissau also add up to WHO's list of the 30 high TB/HIV burden countries in the world. In 2019, 9 out of the 17 West African countries had TB incidence rate of >99 per 100,000 population per year compared to global incidence of 130 per 100,000 population per year.

To reduce this high TB burden, the WHO put in a strategy known as the “End TB Strategy” in 2014 with set targets to reduce the absolute number of TB deaths and TB incidence by 90 and 80% respectively by 2030 and 95 and 90% respectively by 2035 (5). The End TB strategy was unanimously endorsed in May 2014 by all members of the WHO and the United Nations (UN) who proceeded to adopting the UN Sustainable Development Goal (SDGs) in September 2015. The End TB strategy outlines three pillars including; (1) an integrated, patient-centered care and prevention, (2) bold policies and supportive systems, and (3) intensified research and innovation (5). Generally, the control strategy calls for improving diagnostic, intervention, and research tools to facilitate achieving the set targets. Currently, the annual rate of global TB incidence decline is about 2%, and this is far lower than the target of 10% set by the End-TB and SDG strategies. Also, per the End TB strategy, between 2015 and 2020, the total number of TB incidence rate and deaths were expected to have been reduced by 20 and 35%, respectively; however, only 9 and 14%, respectively were achieved.

### The Causative Agent of Tuberculosis

**Figure 1:**
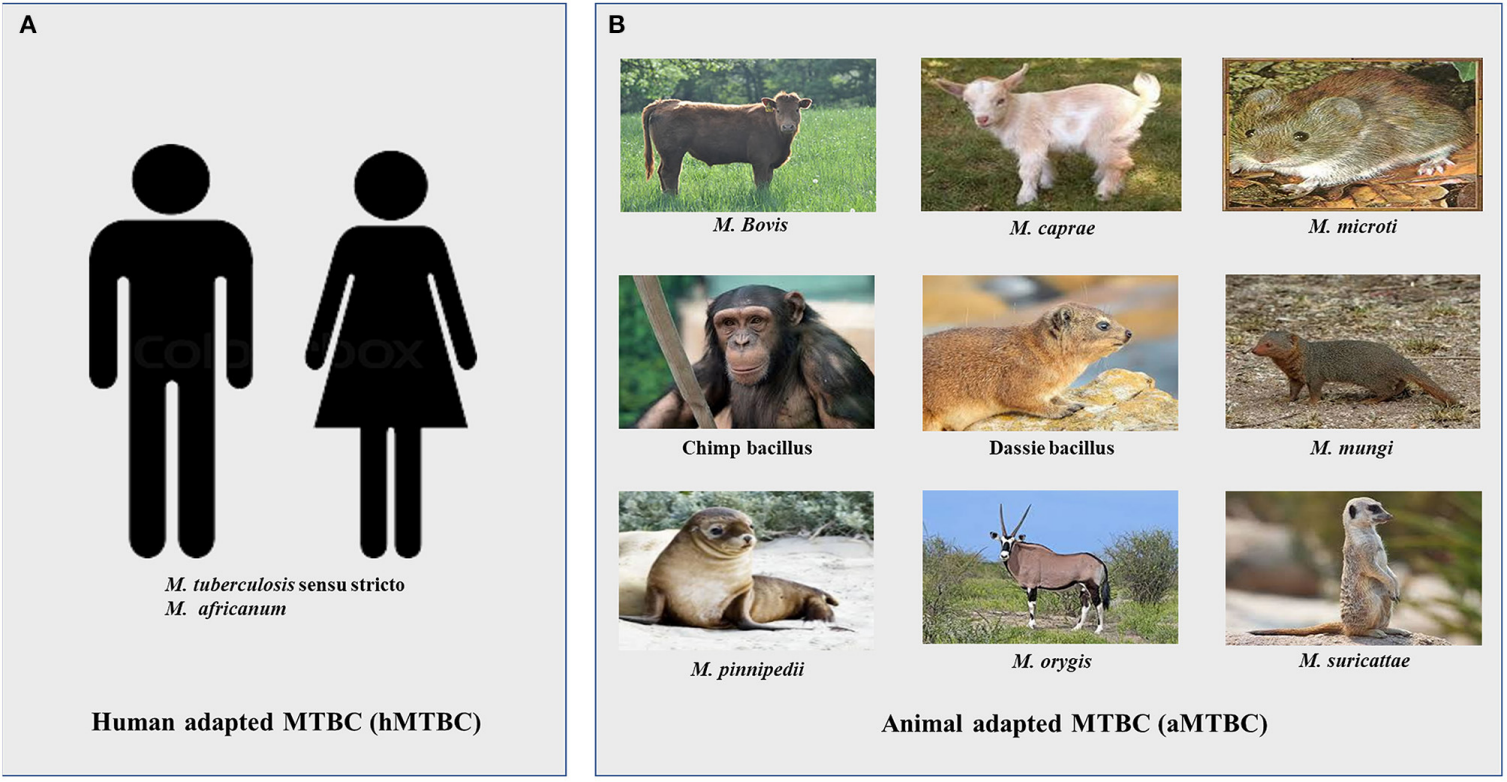
Members of the *Mycobacterium tuberculosis* complex. **(A)** Human adapted *Mycobacterium tuberculosis* complex (hMTBC) **(B)** Animal adapted *Mycobacterium tuberculosis* complex (aMTBC).

Tuberculosis in mammals is mainly caused by 9 genetically related mycobacterial species comprising *Mycobacterium tuberculosis* sensu stricto (Mtb), *M. africanum* (Maf), *M. bovis, M. mungi, M. microti, M. caprae, M. pinnipedii, M. suricattae, and M. orygis* together referred to as the *M. tuberculosis* complex (MTBC) (6–9). The members of the MTBC are intracellular pathogens of mammals whose primary niche is the lungs (6, 10–15). Despite their close genetic relatedness, the MTBC differ in host specificity, although there are occasional cross-species infections (Figure 1). The main human pathogens are Mtb and Maf together referred to as human-adapted MTBC (hMTBC) (6, 12, 13). The animal adapted MTBC (aMTBC) comprising *M. bovis* mainly infects cattle and sheep, *M. caprae* infects goats, *M. microti* infects rodents, *M. pinnipedii* infects sea seals and sea lions, *M. mungi* infects Mangoose, Dassie bacillus infects Dassies, *M. suricattae* infects meerkats, and Chimp bacillus infects Chimpanzees whereas *M. orygis* infects antelopes (6–11, 16).

Maf is endemic in only West-African countries and is responsible for about 50% of TB cases in some of the countries (12, 17–19). Thus, in addition to dealing with the general burden of TB, West Africa has an extra burden to deal with a two-in-one pathogen.

### Control of Tuberculosis and Its Challenges

The traditional methods for TB control depend on vaccination, early case detection of the affected using both clinical and laboratory-based tests followed by antimicrobial treatment of confirmed cases. TB vaccination has however, largely failed the fight against adult TB because the only WHO approved *M. bovis*—bacille Calmette-Guérin (BCG) vaccine administered to over 90% of newborns and in use since 1921 offers mainly protection against disseminated TB in children under 5 years as its efficacy wanes with time (20, 21). The current TB burden could be reduced considerably with a potent vaccine that is able to either induce clearance of latent infections or protect against new infection or both. Thus, early case detection followed by appropriate treatment remains the better option for TB control. Nevertheless, due to severe stigmatization, TB cases delay in reporting to the formal sector case management (22), which contributes to the high case fatality report by some of the countries including Ghana (23).

Laboratory methods used for diagnosis of TB include sputum smear microscopy, nucleic acid based assays, and culture. Smear microscopy prepared directly from sputum specimens is the most widely used test for diagnosing TB in West-Africa, though slowly being replaced by Gene Xpert. Molecular based tests are becoming the preferred test for diagnosing TB as most of them can simultaneously detected drug resistance. Two most widely used assays within the region are the Gene Xpert^®^ MTB/RIF assay (Cepheid, USA) and the line probe assay (LPA) developed by Hain life sciences (GmbH, Nehren, Germany). The Gene Xpert which simultaneously detect TB as well as rifampicin (RIF) resistance directly from sputum has replaced direct sputum microscopy as the primary tool for diagnosing TB in countries like Ghana (3, 4, 24). The LPA which offers a wider spectrum of test including the ability to test for resistance to RIF, isoniazid (INH) fluoroquinolones (FQs), and injectable aminoglycosides (AMG) (GenoType MTBDR*sl*) (3, 25) is more used in second/third level laboratories.

The West-African regions follows the WHO approved case classification for anti-TB therapy. Drug sensitive active TB cases are treated with a 6-month multi-drug therapy which includes INH, RIF, pyrazinamide (PZA), and ethambutol (EMB) (26) which has ~85% treatment success (4). However, there are recent reports of the emergence and high burden of drug resistant TB in the regions (27–30). Drug-resistant TB remains a public health threat. Treatment for drug resistant TB is quite cumbersome requiring at least 9 months (9–20 months) administration of relatively more toxic and expensive drugs such as FQs and AMGs sometimes in combination with linezolid, bedaquiline, and delamanid (4).

Challenges against TB control include socio-economic factors (such as weak health systems, increased urbanization and stigma leading to late reporting); pathogen related factors (such as emergence of drug resistant strains); lack of political will to commit funds and resources for control activities and the HIV epidemic. Lack of cheap but effective sensitive diagnostic tools and limited knowledge of the genome biology of the causative pathogen as well as the transmission dynamics of circulating strains are other equally important factors that hinder TB control. Nevertheless, traditional methods for evaluating TB control programs relies mainly on the number of cases detected and how many were cured, neglecting very crucial questions such as: the duration of infectivity, the frequency of reactivation, and the risk of progression among the infected contacts or the risk of transmission. Various molecular typing tools have been used in molecular epidemiological investigations for studying circulating MTBC strains to aid in TB control (31–33). However, whole genome sequencing (WGS), which has been made possible by the advent and increase in next generation sequencing (NGS) technologies offers the ability to study the genome of MTBC. WGS is crucial for genomic epidemiological investigations which is important for in-depth insight of the nature of pathogens, detecting circulating strains, monitoring resistance, evaluating interventions, and tracking the evolution of the pathogen hence providing a headway to achieve the End TB strategy (5). The SDG and End TB Strategy targets set for 2030 cannot be met without intensified research and innovation. In subsequent sections, we describe in detail some tools for probing MTBC genome, the local epidemiology of MTBC, highlight past and current investigations toward understanding their biology and spread as well as discuss the relevance of genomic studies to TB control in West Africa, the only sub-region that has to deal with a two-in-one pathogen.

## Genotyping Techniques for Epidemiological Study of *Mycobacterium tuberculosis* Complex

Since the early 1990s, several genotyping tools have been proposed for the study of genetic diversity among the MTBC. These tools have been found to be discriminatory enough to distinguish unrelated strains as well as identify closely related strains. Genotyping of MTBC offers several advantages. In particular, it helps to distinguish between new infections and reactivated cases as well as identify predominant genotypes. The classical genotyping methods that have been used to understand genetic diversity among MTBC include large sequence polymorphism (LSP) typing (34), single nucleotide polymorphism (SNP) typing (35), spacer oligonucleotide typing (spoligotyping) (36), insertion sequence 6110 (IS*6110)* restriction fragment length polymorphism (IS*6110* RFLP) typing (37) and mycobacteria interspersed repetitive unit—variable number of tandem repeats typing (MIRU-VNTR) (38) and currently, WGS (39, 40).

### Large Sequence and Single Nucleotide Polymorphism Typing

Large sequence polymorphisms (LSPs) and single nucleotide polymorphisms (SNPs) are phylogenetically robust and stable molecular markers for strain identification. They are unique irreversible events and less prone to distortion by selective pressure due to lack of horizontal gene exchange in MTBC and this makes them less prone to convergent evolution (12, 41). Most importantly, LSPs also known as regions of differences (RDs) (34) have been used to define several discrete strain lineages within the hMTBC specific for different human populations and geographical regions and unravel the evolutionary scenario of ecotypes of MTBC (6, 42). SNPs have also been used to study the biology of the MTBC as a pathogen with very restricted genetic diversity (35). However, these typing tools do not allow the calculation of genetic distances and also cannot completely resolve all deep-rooting branches of the MTBC phylogeny (43).

### Spoligotyping

This tool was developed in 1997 by Kamerbeek et al. (36) and is based on polymorphisms in the clustered regularly interspaced short palindromic repeats (CRISPRs) region of MTBC. Spoligotyping is the most frequently used PCR-based approach for studying the phylogeny of MTBC in high incidence areas. Spoligotyping is simple, cost-effective, and high-throughput with accurate and reproducible results within 2 days. However, it is less discriminatory; it targets only a single genetic locus, covering <0.1% of the MTBC genome. Its direct application on clinical samples without the need for prior culture and easy interpretation and computerized binary (present/absent) data format makes it suitable for molecular epidemiological studies. Direct (from sputum samples) and indirect (using cultured isolates) spoligotyping are both efficient in studying the phylogeny of MTBC, however, in regions with high prevalence of polyclonal infection such as sub-Saharan Africa where all MTBC lineages are present, it is recommended to rule out mixed infection by combining MIRU-VNTR and spoligotyping for more accurate results (44).

### IS6110-RFLP

The first genotyping method developed in the early 1990s by van Embden et al., to be used for strain classification was RFLP based on IS*6110* insertion sequence *(*IS*6110*-RFLP) (37). Initially, considered as the gold standard for transmission studies, this method has been replaced by other methods for various reasons: it is labor intensive, requires high quality DNA, sophisticated and expensive computer software to analyse, experienced personnel of high technical expertise to interpret the results and most importantly, it is not discriminatory enough for strains with 6 or less IS*6110* copy numbers like some strains of *M. bovis*. Nonetheless, it paved the way for an in-depth understanding of the diversity among MTBC before the development of the more recent methods.

### MIRU-VNTR Typing

This is one of the most widely used typing tool and is based on tandem repeat elements dispersed in intergenic regions of the MTBC genomes and copy number diversity (38). Currently, it has become the most reliable and efficient conventional genotyping system for TB transmission studies and has replaced *IS6110*-RFLP. This method has been widely adopted and successfully used in a variety of TB molecular epidemiological studies to trace on-going chains of TB transmission, differentiate relapse from re-infection cases and detect laboratory cross-contamination (43, 45) due to its reproducibility, portability, high discriminatory power, and standardization (33, 43, 45–49). However, it is labor-intensive due to a high number of individual PCRs required and less informative in areas with restricted MTBC lineages (43, 50–52).

### Whole Genome Sequencing as a Typing Method

WGS is increasingly becoming the preferred technique for TB research. WGS determines the complete DNA sequence of an organism's genome at a single time and can provide several answers at a single time, making it the ideal tool for studying the pathogen. With WGS springing up, molecular epidemiology has gradually evolved to become genomic epidemiology. Several studies have applied large-scale WGS to different aspects of TB research; to accurately infer phylogeny (39, 40), to study the biology of the MTBC, and also to study chains of transmission (53, 54) and disease outbreaks (55). Furthermore, WGS has been used to identify drug-resistance associated mutations including; finding mutations compensating for the fitness defect associated with rifampicin resistance (56–58) and rapidly identify drug resistance mutations of an XDR-TB patient (59). These studies demonstrate the potential for future routine applications of WGS in research and genomic epidemiology. However, the use of WGS for large-scale applications especially in endemic areas is limited by its cost and the needed specialized expertise for analyses. A drawback of the current use of WGS in most TB research is that sequencing is mostly carried out on cultured isolates and analyzed using the dominant alleles present without considering within-host diversity. This can however be circumvented by investing in culture-free metagenomics-based approaches.

## Phylogeography of the Mtb and Maf

Similar to other monomorphic bacterial pathogens such as *M. leprae* and *Bacillus anthracis*, MTBC exhibits low DNA sequence diversity and lack of horizontal gene transfer compared to other bacteria (35).

**Figure 2:**
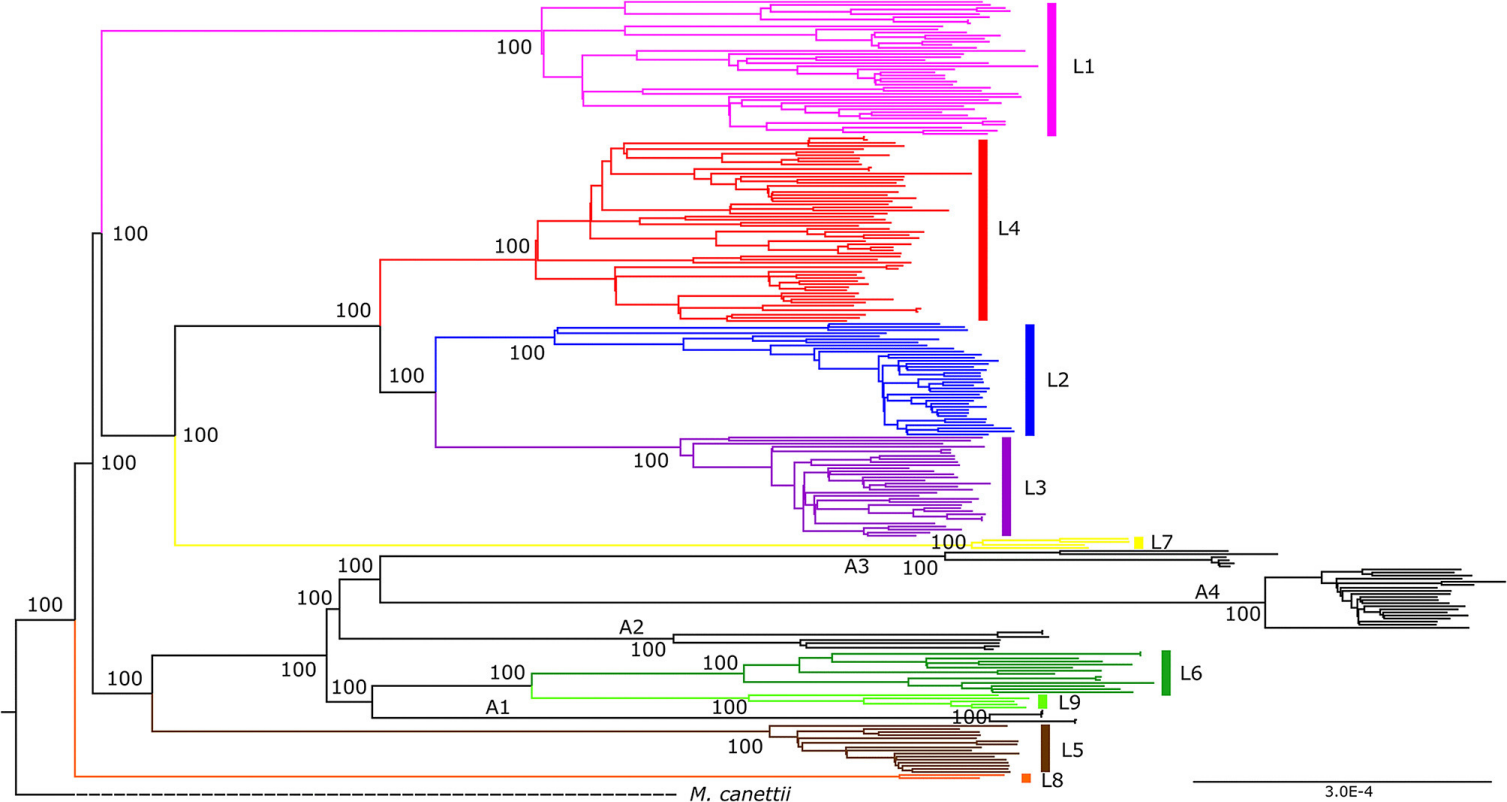
Maximum-likelihood phylogeny of the *Mycobacterium tuberculosis* complex using whole genome sequencing. The phylogeny is rooted on *M. canettii* and bootstrap values shown for nodes. The traditional hMTBC color coding of the first 7 lineages (L1-L7) were used whereas animal adapted members are given the color black. The newly described lineages, L8 and L9 are colored orange and light green respectively [adapted from Coscolla et al. (40)].

The hMTBC were split into 7 main phylogenetic lineages (L) using specific LSPs and SNP markers (42, 60). Lineage 1, L2, L3, L4, and L7 are classified under Mtb whereas L5 and L6 are under Maf. Two additional lineages namely L8 (61) and L9 (40) have been recently identified resulting in 9 phylogenetic lineages of the hMTBC (Figure 2). Nevertheless, L1 to L6 are the main phylogenetic lineages based on the number and proportion of characterized isolates. Using the currently available highly discriminatory genotyping tools, several sub-lineages, and genotypes have been identified among these main phylogenetic lineages of the hMTBC (40, 62–66).

Analyzing the diversity of the hMTBC in conjunction with the country of isolation led to the discovery of different distribution patterns of the various lineages across the globe (12). Whereas, Mtb lineages with the exception of L7 (found at the Horn of Africa) are globally ubiquitous (prominent among them being L4), those of Maf (L5 and L6) are found in West Africa (67). Thus, in addition to dealing with the general burden of TB, West Africa has an extra burden to deal with a two-in-one pathogen. Although Maf is unique to West Africa, its prevalence varies by country. Using molecular genotyping results, the prevalence of L5 increases from West to East and appears highest in Benin (39%) and Ghana (21%), while that of L6 increases from East to West, highest in Guinea Bissau with 51% of smear-positive TB caused by L6 (67).

The observed associations between hMTBC lineage and country of origin under cosmopolitan clinical setting as well as between pathogen lineage and ethnicity within country point to a potential host-pathogen coevolution of the hMTBC and humans (42, 68, 69). Based on this geographical distribution pattern of hMTBC lineages, two groups namely *specialists* (limited to specified geographical locations) and *generalists* (found everywhere) have been proposed (70). L4 is generally described as a generalist lineage whereas L5 and L6 are specialists. Nevertheless, some distinct L4 sub-lineages, are restricted to specified geographical settings including the Uganda, Cameroun and Ghana genotypes (70). It is also possible that the disparate geographical spreading of hMTBC genotypes maybe explained by historical eventualities such as emerging of specific genotypes in regions that later championed colonization and globalization (39, 70, 71). Nevertheless, the potential contribution of biological traits that promote coevolution of specific hMTBC genotypes and certain human populations cannot be understated as evidenced by the San Francisco study which found associations between hMTBC lineage and country of origin of the affected TB patients (42).

## Genome Biology of the MTBC and its Significance Toward the Control of TB in West Africa

The annotated H37Rv genome revealed a genome size of 4.4 Mbp containing ~4,000 genes. The annotated genes include those encoding proteins involved in intermediate metabolism and respiration (877; 22.0%), lipid metabolism (225; 5.7%), information pathways (207; 5.2%), virulence, detoxification and adaptation (91; 2.3%), cell wall and cell processes (517; 13.0%), regulation (188; 4.7%), supposedly conserved hypothetical functions (911; 22.9%) and unknown functions (607; 15.3%). Non-protein-coding regions including, genes encoding stable RNAs (50; 1.3%), insertion elements and remnants of bacteriophages (137; 3.4%) and those rich with PE (Pro-Glu) and PPE (Pro-Pro-Glu) were also identified. The MTBC has extremely high number of genes involved with fatty acid metabolism, especially those associated with β-oxidation of fatty acids which are over 100 (2.5%) compared to 50 (1.2%) genes found to perform the same function in *E. coli* K-12 (72, 73). This large number of the MTBC enzymes dedicated to fatty acid catabolism enhances its ability to thrive in tissues of the infected host, where fatty acids are the major source of carbon (72). Additionally, the presence of glycine rich proteins of the PE (74) and PPE (71) families is unique to the members of the MTBC. Actual functions of these PE/PPE genes are not clearly deciphered. However, similar genes in *M. marinum* have been associated with virulence. Furthermore, antigenicity of some localized PE subfamily of proteins called PGRS (polymorphic GC-rich repetitive sequence) with conserved PE domain followed by Gly-Gly-Ala or Gly-Gly-Asn have been found which underscores the potential implication of these genes in the ability of the MTBC to cause disease.

Comparative genomics analysis of MTBC strains shows little to no evidence of horizontal gene transfer in either hMTBC or aMTBC strains (43, 75–78). This has been attributed to the evolved intracellular adaptation leading to the typical clonal nature of members of this complex compared to most non-mycobacteria and some atypical mycobacteria including *M. abscesses* and *M. avium* (39, 43, 79). Genome diversity within the MTBC thence arise mainly from SNPs comprising insertions, deletions, and substitutions and LSPs including duplication and/or transposition of mobile genetic elements as well as deletion of genetic elements accounting for the different host adaptations, disease phenotypes, and response to interventions (76, 80).

Despite lack of evidence of horizontal gene transfer within the MTBC, recent comparative genomics of the MTBC showed substantial strain diversity among the different members which could have functional implications especially in West Africa where the highest diversity of the MTBC is found (40, 81–87). Comparing the first Maf whole genome sequence (L6 strain GM041182) to H37Rv revealed the presence of a unique sequence RD900 encoding a protein involved with trans-membrane transportation of macromolecules. This sequence is also present in all so-called “ancient” lineages of the MTBC including L1, L5, L6, and L7 but independently lost in all “modern” lineages including L2, L3, L4, and *M. bovis*. Conversely, the Maf genome shares a number of uniquely lost genes with *M. bovis* but not Mtb including genes for biosynthesis of some vitamins arising from pseudogenization. In addition, the L6 genome has an intact copy of the gene (*iniA*) capable of increasing its susceptibility to antibiotics that are not active against Mtb (88, 89). It has also been shown that, all classical hMTBC strains have a conserved *mpt40 gene* which is missing from the genomes of all classical aMTBC an indication of specific host adaptation (90) a discovery that has been incorporated into a rapid nested assay for differential diagnosis of TB (91).

The effects of genomic diversity among bacterial pathogens such as *Escherichia coli, Neisseria menigitidis, Haemophylus influenzae*, B*ordetella*, and *Streptococcus* species are well-documented (92–97). In these species, some strains are more likely to cause invasive disease than others though expression of distinct virulent toxins (93). No such genetic marker (s) has been identified for the MTBC albeit infection by different genotypes results in a range of clinical phenotypes ranging from asymptomatic infection through localized diseased lungs to different forms of disseminated disease (98, 99). However, it is likely that specific MTBC genotypes have distinct genetic traits which influence the immune response elicited by the host, and subsequently the outcome of the host-pathogen interaction (100, 101). This assertion is supported by findings from *in vivo* and *ex vivo* model studies involving different MTBC lineages, indicating that Mtb lineages such as the L2 are more virulent compared to the West-African specific lineages, L5 and L6 (11, 45, 102, 103).

Clinical studies comparing Mtb and Maf (mostly L6), found statistical association between Mtb infection and early progression to active pulmonary TB disease relative to Maf, suggesting that Mtb is more virulent compared to Maf (67, 98, 104–106). Numerous genotype-specific mutations in genes of functional categories such as intermediate metabolism and respiration, cell wall and cell processes, lipid metabolism, regulatory proteins, information pathways and virulence, detoxification, and adaptation could be responsible for the differential presentation of infection by different lineages. Comparative target gene sequence analysis of MTBC lineages found that approximately two-thirds of all SNPs in coding genes are non-synonymous SNPs (nsSNPs) thus underscoring the potential implications of the limited genomic diversity within the MTBC compared to other bacteria (13, 74, 79, 107). Other than SNPs, the reductive evolution of the MTBC involving the genomic deletion of blocks of specific genomic regions including phylogenetic markers can involve blocks of diverse functional genes including but not limited to those associated with immunogenicity and/or host evasion (12, 108, 109). This observation is attributed to the deletion of RD1 a genetic evolutionary event similar to what transpired leading to the generation of the only WHO approved TB vaccine *M. bovis* BCG vaccine from continuous *in vitro* passage of *M. bovis*. The RD1 encodes the *esx1* locus which is responsible for the type VII secretory system driving the secretion of germane T cell antigens ESAT-6 and CFP-10 associated with pathogenicity of the MTBC (110–112).

Recent comparative genomics analysis of MTBC including large number of Maf genomes have revealed many insightful lineage-specific genomic events. For instance, different pairwise SNP distances within lineages (76, 84), different average pairwise nucleotide diversity of annotated genes (84), lineage-specific, and within-lineage accumulation of amino acid mutations (84, 85, 87, 105) some of which could potentially affect the applicability of some control tools (83, 85). Additionally, *in sillico* pan-genome analysis of hMTBC from West Africa identified impaired expression of *mpt64* and *mlaD* genes specifically among L6. Whereas, the *mpt64* encodes the immunogenic protein which is the basis of the *mpt64* rapid TB diagnostics currently in use, the *mlaD* encodes a mammalian cell entry protein. Albeit there was no evidence of impaired expression of *mpt64* among L5, an amino acid mutation I43N within the gene was found specifically among L5. These findings may potentially explain the low sensitivity of *mpt64*-based diagnostics in West Africa where L5 an L6 cause up to 50% of TB (83, 85, 113) as well as the reported slow progression of Maf infection to disease relative to that of Mtb (98). Also, a couple of Maf specific amino acid mutations were found within the *esx-1* secretory system which drives secretion of T cell antigens ESAT-6 and CFP-10 associated with virulence in the MTBC (114). Interestingly, the ESAT-6 and CFP-10 are the backbone of the many interferon gamma release assays used for diagnosis of TB (115) and some potential TB vaccines in different phases of development (116, 117) which could potentially affect the applicability of these interventions in West Africa. Furthermore, essential genes of the MTBC irrespective of lineage were found to be highly conserved and under purifying selection. However, when comparing T cell epitopes, genotypes that are widely distributed such as L4 sub-lineage LAM were significantly diverse compared to T cell epitopes of specialist genotypes such as L5 and L4 Uganda genotypes (40, 70, 84). Unexpectedly, T cell epitopes of L6 which is described as a *specialist* pathogen due to its restriction to West Africa were under positive selection contrary to those of L5 which is also restricted to West Africa. This observation coupled with the statistical association of L5 with ethnicity in West Africa and association of L6 with HIV/AIDS suggest that L5 and L6 may be restricted to West Africa by different biological processes (18, 68). Thus, L6 could potentially be an opportunistic pathogen with an unknown environmental reservoir specific to West Africa (84).

In spite of these indications, exactly how MTBC genomic diversity influences disease progression and presentation as well as the distribution of various lineages and their potential impact on control of TB remain poorly understood. This calls for additional research that seeks to comparatively assess the clinical and ecological implications of MTBC genomic diversity using strain collection that encompass MTBC isolates from every part of the globe toward efficient control of TB.

## MTBC Surveillance and Transmission in West Africa

Surveillance activities geared toward understanding MTBC transmission are necessary to complement conventional control efforts to allow the establishment of good preventive strategies, appropriate therapy, and a better understanding of the pathogen biology thereby contributing to the development of future control tools and ultimately helping eliminate TB. These surveillance activities are specifically needed (1) To correctly identify, characterize and track MTBC lineages/strains; (2) To detect risk factors associated with the disease; (3) To understand MTBC person-to-person transmission dynamics, which has been studied extensively in developed countries of North America and Europe as well as other parts of the world and has been useful for identification of outbreaks as well as most at risk groups (33, 48, 118–120) for targeted control activities and; (4) To track TB strains among recurring TB patients and provide indications of the cause of secondary case source (121–123), for appropriate treatment, evaluation of performance and epidemiology (32, 124). To effectively control TB in West Africa, it is therefore paramount to undertake such investigations in a population-based scale which will contribute to knowledge on factors that enhance spread of the disease in the sub-region.

The molecular typing tools discussed earlier have not only been used to study MTBC biology but also for surveillance purposes through molecular epidemiological investigations. Although the typing tools possess varying discriminatory power (32, 125–129), they have been used widely in advanced countries to help monitor MTBC spread especially among prisoners and cross country travelers (130–133). In West Africa, findings from surveillance studies have revealed in-depth knowledge of the varying distribution of Mtb and Maf (134, 135) and has called for further studies to investigate their transmission dynamics within respective geographical areas. This has a great public health value considering that members of the MTBC do not all have the same disease phenotype hence the need to survey to obtain knowledge of circulating genotypes. Some interesting observations has been made over the years from surveillance activities conducted in West Africa. For instance, as identified elsewhere (136–141), an association between Beijing strains of L2 and drug resistance has been reported in Benin including the identification of a possible streptomycin-resistant Beijing outbreak (142, 143) and similarly, through surveillance activities conducted in Ghana, the Ghana genotype of L4 has been linked to drug resistance (19, 144). The spread of difficult-to-treat drug resistant strains are also monitored through surveillance activities (31, 132, 145, 146). Until recently, drug resistant clones were thought to be less fit and less likely to transmit from person to person; however recent surveillance studies in Ghana, Nigeria, and other parts of the world have documented evidence of transmission of both INH resistant strains and MDR even though not involving large clusters (53, 118, 147, 148). There is therefore the need to identify and control such difficult-to-treat drug resistant clones to stop their spread through surveillance activities. WHO currently supports the inclusion of NGS to provide detailed information on drug resistance across multiple gene regions directly from sputum specimens, however the WHO is yet to review and approve current protocols (4). Also, through surveillance activities, zoonotic TB spillover has been observed in West Africa with indications that individuals who are in direct constant contact with livestock and/or dairy products are at major risk of contracting zoonotic TB (19, 149, 150). Finally, among others, surveillance activities in the sub-region has led to the observation that the West African restricted Maf specie has reduced transmissibility (45), has decreased sensitivity to some available diagnostics (83), has poor progression to active disease (98, 151), has poor treatment outcome (152, 153) and has been found to be associated with some endemic ethnic groups (18, 68).

With the advent of new genotyping techniques, surveillance activities are made more meaningful through MTBC transmission studies. The MIRU-VNTR typing tool has over the years been widely adopted and together with epidemiological data used in a variety of TB transmission studies for the detection of recent TB transmission and outbreaks due to its portability, standardization, reproducibility, and high discriminatory power (31, 33, 45–49, 154, 155). However, only few West African studies have employed this tool for transmission studies to identify genetic and geographic TB clusters (45, 105, 135). The discriminatory power of this largely adopted MIRU-VNTR typing tool may not be sufficient to distinguish unrelated strains for some geographical settings (126) especially in West Africa with the most diverse MTBC lineages, hence for TB transmission studies it is recommended that WGS, which is by far the ultimate tool for strain differentiation, be used to make decisive conclusions. Data generated from WGS offers the ability to accurately identify recent TB transmission and also to trace the direction of transmission between epidemiologically linked cases (156). However, this tool has not been readily utilized for MTBC transmission studies in West Africa probably because of the huge cost and expertise needed to analyze the generated data. This may not be a problem in the near future as WGS is gradually becoming less expensive. The recent increase in NGS technologies coupled with the competition among available sequencing platforms and the availability of simpler data analysis tools have made WGS an attractive molecular tool used in many surveillance and transmission investigations. Globally, the first report of an effective use of WGS for MTBC transmission investigation was in 2011 from Vancouver which involved the delineation of two unrelated transmission events among a cohort of drug users having identical MIRU-VNTR profiles following which it has been used in a large array of studies (148, 157–161). WGS has been useful in detecting unsuspected outbreaks hence it should be used not only as a research tool but as a surveillance tool to aid in providing the necessary guided steps to track, monitor, and control MTBC strains.

There exist varying reports on the transmissibility of members of the MTBC (45, 98). However, limited surveillance activities have suggested that Mtb transmits better than Maf (45, 162). This is probably because Maf is thought to be attenuated compared to Mtb (67, 113, 163). Although it is argued that Mtb is fitter than Maf, and with time it will outcompete the Maf population, this may not be true as two studies from the Gambia and Ghana have proven otherwise by observing a constant prevalence of Maf over a period of 7 and 8 years, respectively (19, 152). However, decline in Maf prevalence have been reported in a number of studies from Guinea-Bissau, Côte d'Ivoire, and Cameroon (164–168). Some of these studies employed biochemical means for classification and perhaps might have misclassified the strains. There is therefore the need to invest more resources in using higher resolution tools such as the WGS for MTBC surveillance and hence transmission. Understanding MTBC transmission will contribute to knowledge on factors that enhance the spread of the disease, which is useful for developing preventive interventions and may have implications for the development and deployment of new TB vaccines and diagnostics. For instance, hotspots of TB transmission identified through a recent study in Ghana (53) offered the TB control program to direct some of their limited resources to targeted population groups for increased awareness and enhance screening activities. Also, using WGS, there is reported evidence of person-to-person transmission of Maf lineage 6 strains from Mali (87) confirming the propensity of the Maf species to also transmit (45).

Finally, through molecular surveillance activities, TB relapse are now correctly defined. Formally, all individuals who get a secondary episode of TB are referred to as having relapse. By definition, a disease is said to have relapse if the old infection bounces back. This is however not true for all secondary episodes of TB knowing that some individuals do come back with totally different strains compared to the previously infecting strain; such cases are technically referred to as exogenous re-infections rather than relapse. A secondary TB episode can be referred to as relapse only if the previous infecting strain is the same as the current one. Thankfully, current MTBC genotyping tools make it possible in most instances to distinguish between relapse and re-infection. This is possible because of the assumption that if strains from both episodes are genotypically/gnomically indistinguishable, it suggests relapse whereas distinguishable strains suggest re-infection. Predominance of relapse over re-infection indicates high-quality public health practices and a low risk of local transmission. Many studies have employed genotyping tools like MIRU-VNTR, IS*6110* RFLP, and WGS technique to explore relapse and re-infection among TB patients (121–123, 169–174). However, very few of these studies originated from West Africa. Majority of the few previous studies conducted in Africa have only employed large DNA-sequence based typing assays (ie. MIRU-VNTR and IS*6110* RFLP) which can potentially be confounded by convergence evolution. This means that established relapse cases may not actually be relapse events hence we advocate for the use of WGS which is more robust and relatively free from convergence evolution. Using WGS, it was possible to accurately detect relapse from a Ghanaian cohort; this study identified a couple of individuals who were previously infected with drug sensitive strains but later had TB recurrence harboring drug resistant strains (121). This shows the possibility to track such recurring cases and highlights the need to foster genomic epidemiology to aid early detection of drug resistance emergence to provide an effective TB control. Such surveillance activities carried out using WGS data are currently not absolute and has a few limitations as the common practice has been to make judgements based on sequencing one isolate per individual at each time point neglecting the possibility of within-host bacteria diversity. However, it is possible to detect mixed infections (175). It is worth noting that the confidence in differentiating relapse and re-infection can be reduced without considering the various bacterial populations that may exist at a given time point. We acknowledge that such within-host diversities do exist, and current and future studies should consider it in their investigations.

## Mechanism of Drug Resistance Emergence and Evolution

The control of tuberculosis has been hampered by the emergence of drug resistance globally which threatens to make TB untreatable (4, 176). Drug resistant TB in the Africa region has been a major threat to the achievement of the goals of WHO's End TB Strategy and the SDGs in the region (4). The spread of these resistant strains mimics the pre-antibiotic era. According to the 2020 Global TB Report, about 0.5 million individuals developed rifampicin-resistant TB (RR-TB) and 78% of this number were confirmed as multi-drug resistant TB (MDR-TB) cases (4). The drug resistance TB cases contributed 3.3% of all new TB cases and 17.7% of previously treated people (4).

The threat posed by drug resistant TB strains can be mitigated by gaining a better understanding of the mechanism of the emergence of these strains and their evolution. With the recent advances in bacterial genomics using NGS, the molecular mechanisms of emergence, fixation, and transmission of drug resistant TB are being unraveled (177). However, there is the need to further assess the complexity of the emergence of drug resistant strains that have become a major challenge to the control of the disease. Comparative genomic studies have also shown that strain diversity could also be a major factor heightening the threat of TB drug resistance (40, 178, 179). This section seeks to throw more light on the advances made toward deciphering the mechanisms of drug resistance especially among the West African genotypes of MTBC and to provide new directions for future studies.

**Figure 3:**
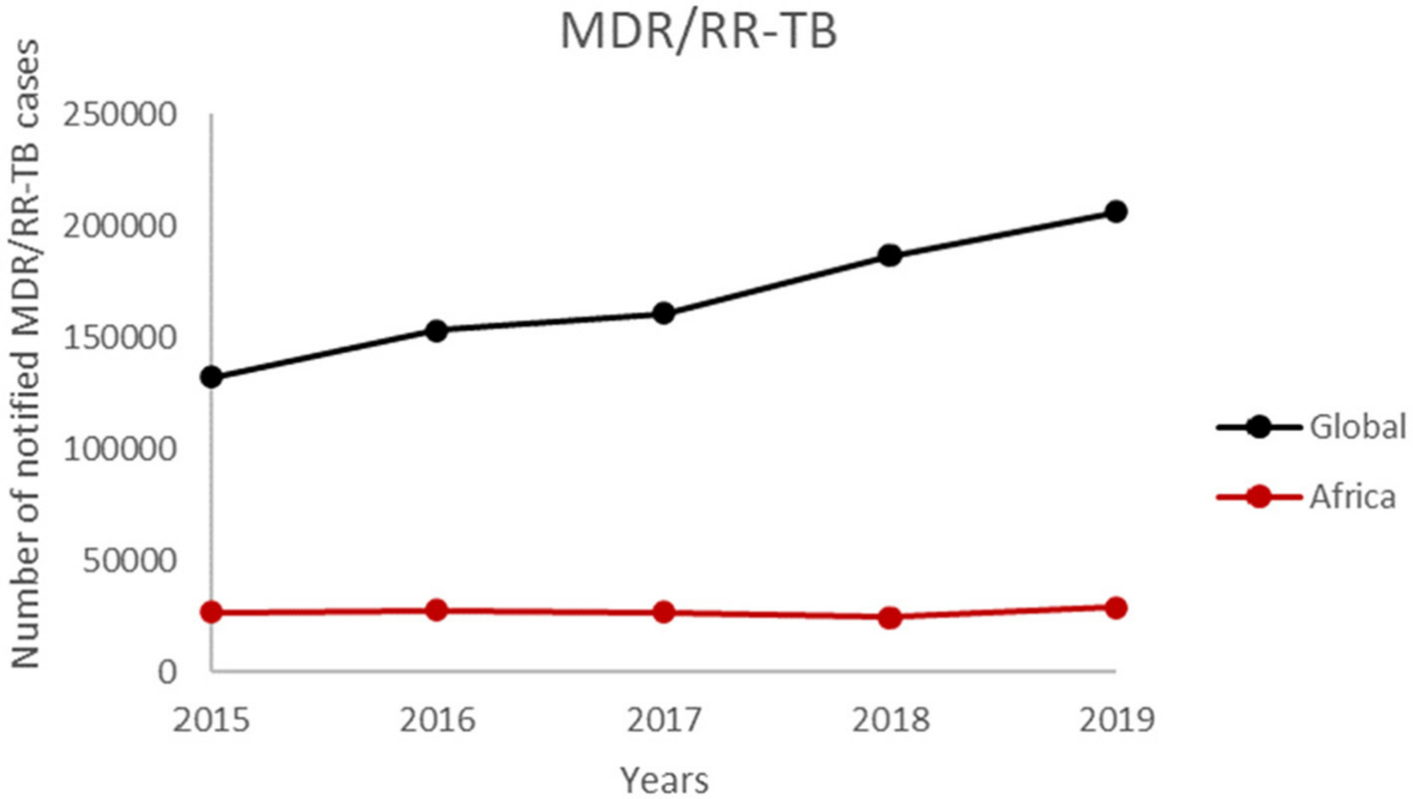
The number of notified MDR/RR-TB cases by WHO member states from 2015 to 2019.

There is paucity of data on TB drug resistance in sub-Saharan Africa which has always called for active surveillance to determine the true burden of DR in West Africa and Africa as a whole (178). The WHO initially reported the burden of drug resistance in West Africa based mainly on projected estimates. An active surveillance conducted by the West African Network of Excellence for Tuberculosis, AIDS and Malaria (WANETAM), on isolates from 2009 to 2013 showed an unexpectedly higher MDR-TB of 6% for new TB cases as compared to the 3.5% WHO estimate in 2013 (180). Although the WHO estimated 20.5% MDR-TB for retreatment cases (classified as patients who have been previously treated for TB and have reported again with the disease), the network reported 35% for the eight West African countries (28, 180). In another study that screened isolates from the eight West African countries from 2012 to 2014, the authors also reported the same 6% MDR-TB for new cases and 34% for retreatment cases (30). This shows the prevalence of drug resistance in West Africa has been fairly constant and multi-drug resistance has become an emerging health challenge in West Africa. The same trend is seen in the WHO Africa region where the number of notified MDR/RR-TB cases have been constant over a 5-year period (Figure 3).

Several studies have linked specific lineages/sub-lineages with drug resistance (179, 181). A typical example is the Beijing sub-lineage of MTBC which is associated with MDR-TB in Asia (182, 183). The propensity for some of MTBC genotypes to harbor resistance toward anti-TB drugs have been reported in the sub-region. For instance, the Ghana sub-lineage of L4 which is mainly restricted to West Africa has been found to be associated with drug resistance in Ghana (144). Comparative genomics analysis of hMTBC from Ghana revealed that INH resistant Mtb and Maf were significantly associated with *katG* and *inhApro* mutations, respectively (144). In a separate study, a univariate analysis revealed that L6 was less likely to be associated with INH resistance (18). In support of this, a most recent comparative genomics analysis of Maf has shown that L5 has a high genetic inclination toward development of drug resistance compared to L6 (40). West Africa is unique in its MTBC genotypes and evolution of drug resistance which calls for further molecular investigation. Although a number of the resistant isolates reported in the sub-region were sequenced and analyzed for the presence of mutations associated with drug resistance, there is still a lot that were only characterized by phenotypic methods (18, 28, 30, 144).

**Figure 4:**
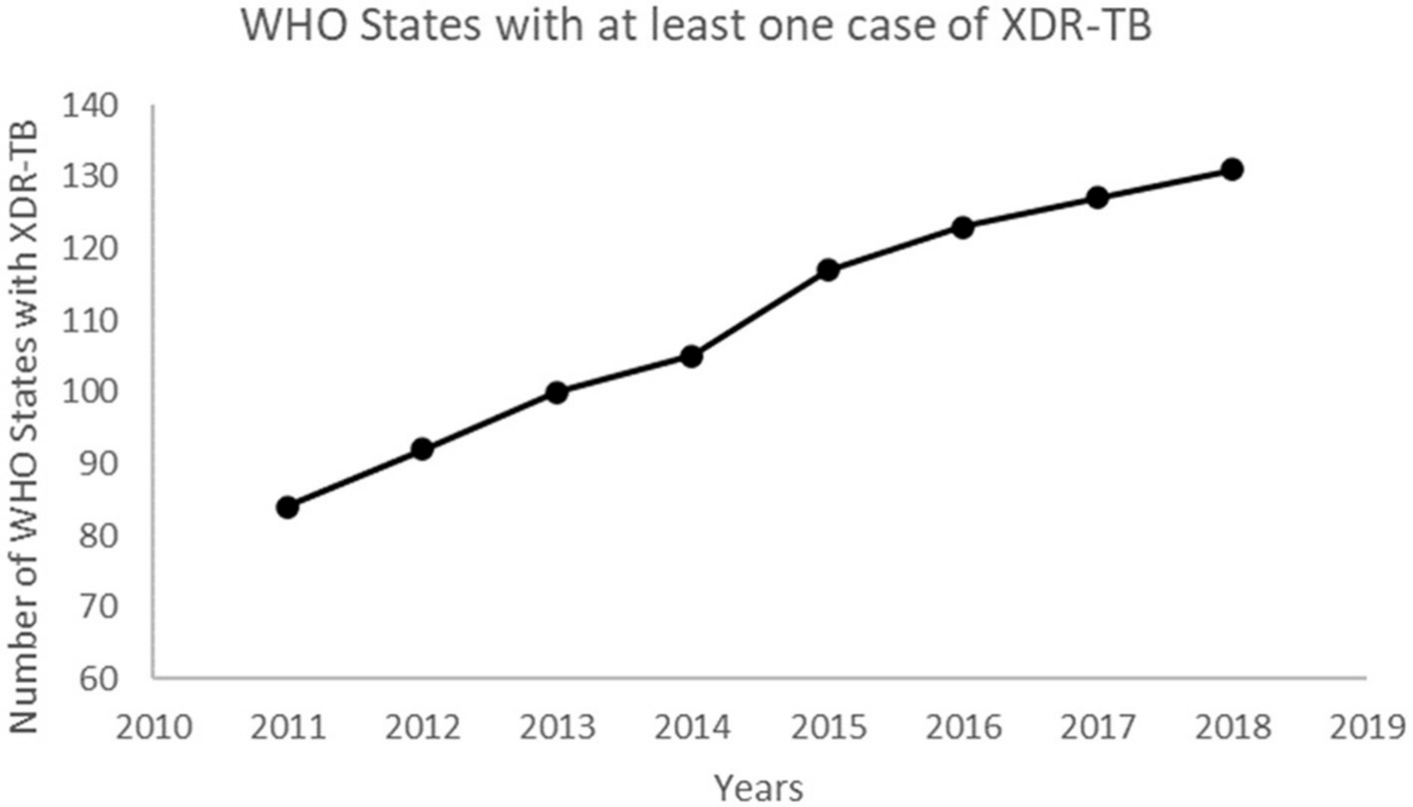
The number of WHO member States with at least one confirmed case of XDR-TB.

**Figure 5:**
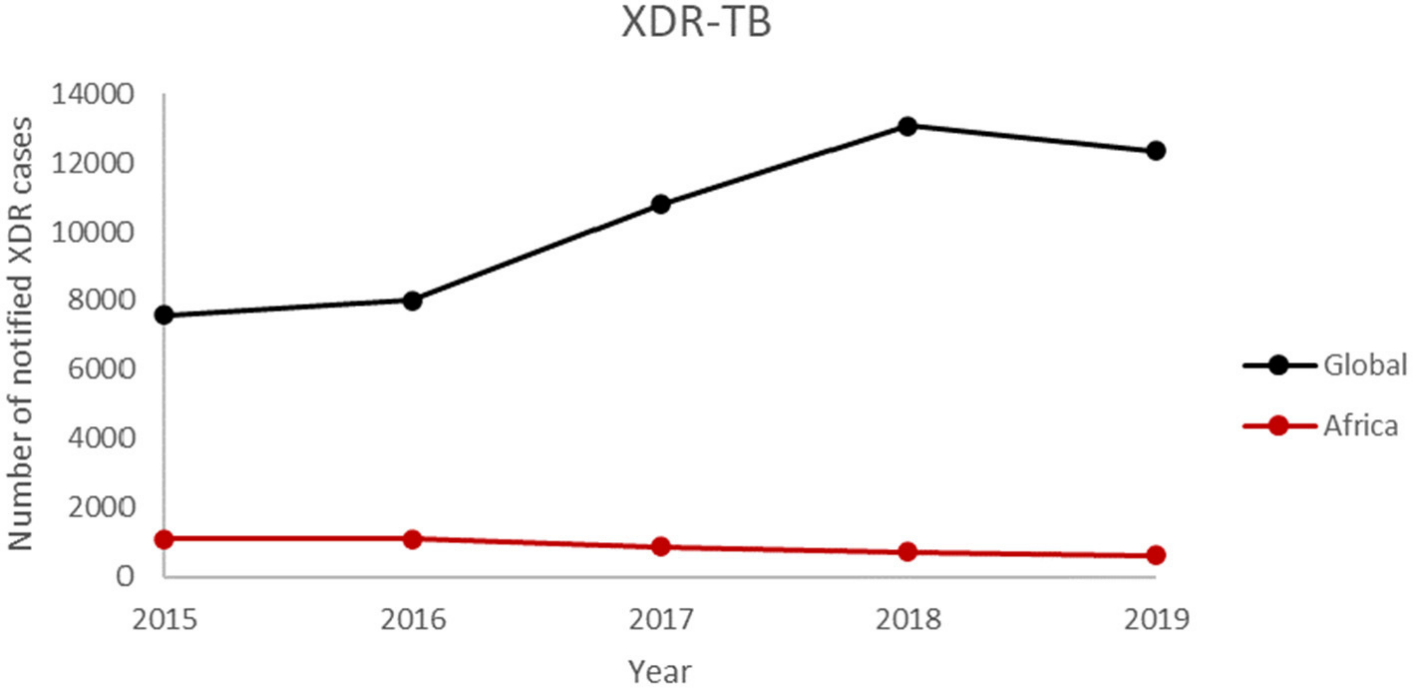
The number of notified XDR-TB case from 2015 to 2019.

The number of WHO States with at least one reported case of XDR-TB has been increasing over the years (Figure 4). This confirms the spread of XDR-TB globally. The number of reported XDR-TB cases has been on the rise with that of Africa on a slight decrease (Figure 5). The DR picture for Africa might look different for different parts of the region.

The WANETAM reported the circulation of pre-XDR-TB, which is MDR-TB with additional resistance to either a fluoroquinolone or an aminoglycoside, among retreatment cases in all the eight countries in 2016 without any record of XDR-TB. Interestingly, only Ghana and Togo had reported pre-XDR TB among new cases. It was not surprising when Ghana reported its first XDR-TB case in 2018 (29). Togo, together with Burkina-Faso and Niger had already reported at least one XDR-TB case in 2011 (184). This calls for more genomic studies in West-Africa to understand the evolution and spread of DR-TB in West-Africa.

## Future Perspective

Despite these evidence of genomic diversity among the MTBC supported by phenotypic data including variable outcome of TB infections, differences in macroscopic morphology, niacin production, and inhibition by pyrazinamide (185, 186), most research supporting global interventions are largely based on limited and biased collection of isolates. The presence of a unique TB causing bacteria restricted to West-Africa makes it imperative for more genomic studies in the sub-region to improve the understanding of the biology of Maf and the functional implications of genomic diversity between lineages of Maf and that of Mtb.

As has been detailed above, genomics and bioinformatics, though relatively new in biomedical disciplines, they are very useful tools for diseases surveillance and have a role to play in the control of infectious diseases including tuberculosis. WGS of MTBC can be used in routine care settings for species identification, determination of drug resistance profiles and to complement epidemiological source investigation. Although few research works have used these tools in West-Africa to date, they are increasingly being used especially in universities and research institutions. Nevertheless, due to cost and infrastructural as well as expertise demands, its usefulness has not been felt in public health intervention programs. Some implementation challenges include funding for infrastructure as well as expertise for WGS in many research settings and even greater challenges in the context of the clinical settings. Major efforts need to be made in building human capacity as well as infrastructure investment in national public health reference laboratories to improve health care in high burdened countries. Moreover, the cost of WGS is coming down drastically and there are available simplified/portable platforms such as the minion that is field friendly and less expensive. The establishment of WGS in routine settings such as regional and national public health reference laboratories of the national health system will need to be done in a way that is relevant to the local health priorities. It is anticipated that current capacity building being championed by programs such as the African Centers of Excellence being financed through World Bank Loans, DELTAS financed by Wellcome Trust through the African Academy of Sciences will enhance implementation of WGS as part of routine health service surveillance activities, which improve health delivery in West African countries such as Ghana. This is evidenced in the contribution of some of the centers in the sequencing efforts of SARS-CoV 2 within the region (187).

The highest incidence and impact of antimicrobial resistance (AMR) is experienced in resource-poor settings (188). Underlying factors promoting AMR include misuse of antimicrobials, lower access to alternative antibiotics and the prevalence of multidrug-resistant bacterial strains (189). The slow growth nature of the MTBC negatively impacts the use of culture for routine surveillance of DR, hence, making molecular detection the better option. Routine and systematic surveillance of AMR infections is key to inform policy decisions and public health interventions to counter AMR. WGS and targeted sequencing offer the opportunity for the identification of the causative pathogen, to understand the genetic basis of resistance, as well as pathogen evolution and population dynamics at different spatial and temporal scales. It also offers the opportunity to probe the whole genome to detect not only susceptibility to a single antibiotic but multiple antibiotics at the same time. It is envisaged that potable sequencing platforms will be established at least in national and supra national reference laboratories within the region to support the DR surveillance efforts.

The use of WGS has a great prospect toward improved individual case management of infectious and non-communicable diseases. Advancement in simplified, high-throughput genomic technologies will in future assist West-Africa to sequence the whole exome or genome of a person at a price that is affordable for some health-care systems. More services based on these technologies will enhance host-directed therapies appropriate to individual patients with probing limited to analysis of specific (sets of) genes of clinical value (190, 191). WGS in future will be useful in West-Africa for evaluation of interventions such as vaccination for preventive policies through enhanced assessment of disease and drug resistance transmission dynamics. Furthermore, it will improve pathogen detection, especially with the emergence of new and un-culturable infections as well as biological risk prediction.

## Author Contributions

All authors listed have made a substantial, direct and intellectual contribution to the work, and approved it for publication.

## Conflict of Interest

The authors declare that the research was conducted in the absence of any commercial or financial relationships that could be construed as a potential conflict of interest.

## Publisher's Note

All claims expressed in this article are solely those of the authors and do not necessarily represent those of their affiliated organizations, or those of the publisher, the editors and the reviewers. Any product that may be evaluated in this article, or claim that may be made by its manufacturer, is not guaranteed or endorsed by the publisher.
